# Building Keypoint Mappings on Multispectral Images by a Cascade of Classifiers with a Resurrection Mechanism

**DOI:** 10.3390/s150511769

**Published:** 2015-05-21

**Authors:** Yong Li, Jing Jing, Hongbin Jin

**Affiliations:** School of Electronic Engineering, Beijing University of Posts and Telecommunications, Rd. Xitucheng 10#, Beijing 100876, China; E-Mails: jingheiieh@bupt.edu.cn (J.J.); jinhongbin@bupt.edu.cn (H.J.); qiaowei@bupt.edu.cn (W.Q.)

**Keywords:** multispectral, cascade structure, resurrection mechanism

## Abstract

Inspired by the boosting technique for detecting objects, this paper proposes a cascade structure with a resurrection mechanism to establish keypoint mappings on multispectral images. The cascade structure is composed of four steps by utilizing best bin first (BBF), color and intensity distribution of segment (CIDS), global information and the RANSAC process to remove outlier keypoint matchings. Initial keypoint mappings are built with the descriptors associated with keypoints; then, at each step, only a small number of keypoint mappings of a high confidence are classified to be incorrect. The unclassified keypoint mappings will be passed on to subsequent steps for determining whether they are correct. Due to the drawback of a classification rule, some correct keypoint mappings may be misclassified as incorrect at a step. Observing this, we design a resurrection mechanism, so that they will be reconsidered and evaluated by the rules utilized in subsequent steps. Experimental results show that the proposed cascade structure combined with the resurrection mechanism can effectively build more reliable keypoint mappings on multispectral images than existing methods.

## 1. Introduction

Multispectral imaging techniques have been applied in a variety of fields, including civilian surveillance, intelligent navigation, automatic target recognition, *etc*. For example, researchers wish to analyze with images geographic and geological change before and after a devastating earthquake, to help conduct a better reconstruction. These images are often taken at different times, from different views and/or with different spectral light. They provide complementary information to the visible spectrum [1] and make image analysis more reliable. To effectively utilize them, an accurate registration is necessitated to account for the misalignment between images, so that all images of a scene can be aligned to a common coordinate. Then, a fine-fused image can be generated as if it were taken by one camera.

During the last few decades, many methods have been proposed to align multispectral images. A category of commonly-used methods addresses the image registration by building keypoint mappings. These methods generally include three steps: detecting keypoints, calculating descriptors and building keypoint mappings with the descriptors associated with the detected keypoints. Typical keypoints and descriptors include the Harris corner [2], SURF [3], SIFT [4], MSER [5], WLD [6], *etc*. These descriptors are often devised to be invariant to small scaling, rotation and even affine transformation on single-mode images. Morel and Yu [7] propose ASIFT, which is fully affine invariant. It simulates all image views obtainable by varying the two camera axis parameters and covers the other four parameters using SIFT. Cai *et al.* [8] then further proposed a perspective scale invariant feature transform (PSIFT) using homographic transformation to simulate perspective distortion. To increase the number of keypoints, Park *et al.* [9] proposed using higher-order scale space derivatives, ∂^2^*L*(*x,y*,σ)/∂(σ^2^, ∂^3^*L*(*x, y*, σ)/∂σ^3^, ∂^4^*L*(*x, y*, σ)/∂σ^4^, and then extracted the extrema in the high-order scale space.

However, if the two images to be registered are taken by different spectral light, e.g., thermal infrared and visible light, the ratio of correct keypoint mappings established with SURF and SIFT will dramatically decrease, resulting in a significant decrease of registration performance. To adapt the descriptors designed for single-mode images (e.g., SIFT) to multispectral images, many techniques have been proposed, including NG_SIFT (NG, normalized gradient) [10], SAR_SIFT [11] and MIND [12]. NG_SIFT utilizes the normalized gradients around keypoints for describing the local pattern to achieve the invariance against non-linear intensity changes between multispectral images. It outperforms the original SIFT on the multispectral images of a structured scene. SAR_SIFT proposes a new local gradient pattern around keypoints, in which the orientation and magnitude are robust against the speckle noise. SAR_SIFT gives a higher ratio of correct keypoint mappings than the original SIFT on multispectral images. Mainali *et al.* [13] proposed the D-SIFER scale-invariant feature detection algorithm using the 10th order scale-space optimal Gaussian derivative filter. D-SIFER was validated on hyperspectral images and was shown to perform better than SIFT and SURF.

Aguilera *et al.* [1] proposed EOH (edge of histogram) by borrowing the idea from MPEG-7 descriptors [14]. EOH considers only the edge pixels for computing descriptors (also, see [15] ) and does not utilize the gradient magnitude of a pixel weighing its contribution. Unlike other descriptors, EOH generates 4 × 4 × 5 descriptors, with 4 × 4 blocks for the neighboring window around keypoints, five orientation bins in each block, including 0°, 45°, 90° and 135°, and a non-direction bin. Similar to EOH, Bingjian *et al.* [16] computed descriptors with edge points only, but the local window around a keypoint is partitioned into 17 bins in a log-polar coordinate.

Existing descriptors for multispectral images perform well when the spectral distance is small. Spectral distance here refers to the distance/difference of two imaging wavebands. For example, the spectral distance between Band 1 Visible (0.43–0.45 μm) and Band 10 TIRS 1 (10.6–11.19 μm) in Landsat 8 is approximately 10.6 – 0.45 = 10.15 μm. Since the common information between multispectral images decreases with the increase of spectral distance, the keypoint mappings built solely with the matching ability of the associated descriptors often contain a high ratio of incorrect mappings. Effectively representing common information between multispectral images is not an easy task. Among commonly-used information is the gradient pattern (e.g., SIFT), edge points, *etc*. Some similarity metrics can be treated as a variant of common information, e.g., linear correlation and mutual information (MI) [17]. Correlation assumes the linear relationship between image intensities, which often does not hold for multispectral images [18]. In fact, gradient computation is a linear operation of image intensities, so the linear relationship is the foundation of SIFT descriptors. MI utilizes the statistical dependence between intensities. However, it is sensitive to keypoint positions and excels at finer registration, e.g., [19].

Given the matching ability of descriptors, an essential problem is now to remove the incorrect keypoint mappings. Techniques, such as RANSAC [20] and CIDS (color and intensity distribution of segment) [21], have been proposed to identify the correct mappings from the initial set built with the associated descriptors. Gong *et al.* [19] proposed a coarse-to-fine registration scheme on multispectral images by combining SIFT with RANSAC and the mutual information (MI) [17]. However, most of the existing approaches to removing incorrect keypoint mappings apply only a one-pass process, e.g., RANSAC [20], CIDS [21], BBF (best bin first) [22] and FSC (fast sample consensus) [23]. The problem with these approaches is that when the ratio of correct keypoint mappings is low, the rate of correct mappings being misidentified as incorrect (and the reverse) will be very high. Consequently, the set of the keypoint mappings preserved by these methods still contains a large percent of incorrect mappings, and also, some correct mappings are removed by mistake.

Observing this and motivated by the boosting technique [24] in the field of object detection, we abandon the idea of deciding on keypoint mappings in only a one-pass process and propose a cascade structure with a resurrection mechanism. The cascade structure is composed of multiple steps (different approaches) to construct keypoint matchings. The resurrection mechanism will evaluate the quality of each keypoint matching and assign a score to it. The score is to be updated in the next step according to its matching quality at that step.

Each step of the cascade structure employs a “loose” rule, so that only the keypoint mappings that score very low for this rule will be removed, and the other mappings remain to be undetermined or temporarily considered as correct mappings. The remaining mappings are to be evaluated in the following steps of the cascade structure by the resurrection mechanism. Since it is almost certain that some correct keypoint mappings are mistakenly removed at every step, the resurrection mechanism allows for a fraction of wrongly-removed keypoint mappings to be reconsidered and evaluated in the subsequent steps. Alternatively, the resurrection mechanism can be thought of as a compensation for the drawback of the rules utilized in the cascade structure, as this “gets back” some of correct mappings that are removed according to the rules.

The rest of this paper is organized as follows. Section 2 discusses the proposed resurrection mechanism. Section 3 discusses the proposed cascade structure that is comprised of four steps. Section 4 presents the experimental results, and Section 5 concludes this paper.

## 2. The Resurrection Mechanism

The repeatability of keypoints under modality change is low, and to obtain more correct keypoint mappings, we propose a resurrection mechanism for reducing the number of mis-removed correct mappings due to repeatability insufficiency. This idea arises from the observation that some correct keypoint mappings are often misjudged by any keypoint matching method. In the resurrection mechanism, a score is assigned to every keypoint mapping based on the matching quality assessed with the method applied in one step of the cascade structure. The score is to be updated in the next step according to the matching quality assessed with the method applied at that step. Through the resurrection mechanism, each keypoint mapping will be evaluated by different matching criteria. This gives the chance for the misjudged correct keypoint mappings to be reconsidered and evaluated in subsequent steps; otherwise, they would have been removed permanently. To understand the resurrection mechanism, a confidence grade *cg* is defined to indicate the matching quality of each keypoint mapping at one step in the cascade structure. According to the matching quality, *cg* is set to 0, 1, 2 and 3, respectively.

*cg* = 0 means that a keypoint mapping is of the worst quality, and it will be removed and never be considered. *cg* = 1 means that the quality of a keypoint mapping is not high enough at the current step, but may possibly be high enough at the next step. If *cg* = 2 or 3, the keypoint mapping will successfully go through the current step to the next step. At the next step, *cg* is updated for each keypoint mapping, and the keypoint mappings with *cg* = 0 will be removed. The keypoint mappings with *cg* = 1, 2 or 3 will be passed to the next step. In the last step, the keypoint mappings with *cg* = 2 or 3 are defined to be the matched keypoints from the proposed cascade structure.

Let *cg_p_* denote the *cg* value of a keypoint mapping at the previous step. Let *cg_t_* denote the *cg* value determined only by the method applied at the current step. Then, the score *cg_c_* at the current step can be assigned (updated) as follows,

(1) $$cg_{c}=\{\begin{array}{ll} cg_{t} & cg_{p}>1\\ cg_{t}-1 & cg_{p}=1 \end{array}$$

Note that the keypoint mappings with *cg_p_* = 0 have already been removed at the previous step.

**Figure 1:**
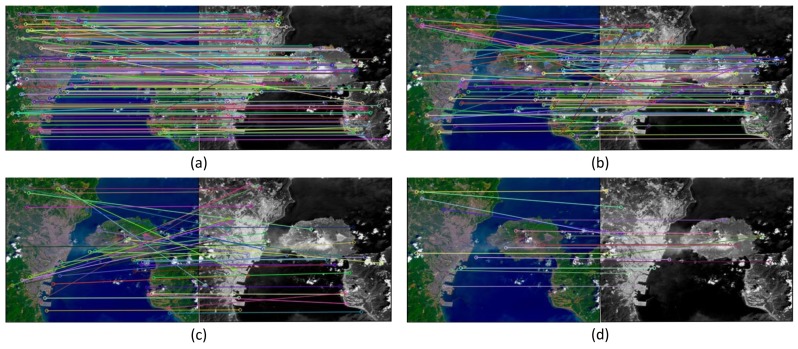
The example of resurrection. (**a**) The mappings whose *cg* is three in Step 1; (**b**) the mappings whose *cg* is two in Step 1; (**c**) the mappings whose *cg* is one in Step 1; (**d**) the mappings resurrected from (c) in Step 2.

Figure 1 gives an example showing the functionality of the resurrection mechanism. We utilize the BBF in Step 1 and the CIDS in Step 2 in the proposed cascade structure. In Step 1, keypoint mappings are divided into three levels according to their scores. Figure 1a shows 350 keypoint mappings whose *cg* is three, including 270 correct mappings with a correct ratio of 71%. Figure 1b shows 166 mappings whose *cg* is two, including 68 correct mappings with a correct ratio of 41%. Figure 1c shows 119 mappings whose *cg* is one, including 22 correct mappings with a correct ratio of 19%. This shows that a higher *cg* stands for a greater probability that a keypoint mapping is correct. However, there are correct mappings in the pending keypoint mappings, as shown in Figure 1c, which would have been determined to be incorrect if only one step were utilized. In Step 2, 18 mappings are resurrected from those pending mappings, as shown in Figure 1d, and there are 13 pairs of correct keypoint mappings “recovered” with the resurrection mechanism.

## 3. The Proposed Cascade Structure with the Resurrection Mechanism

**Figure 2:**
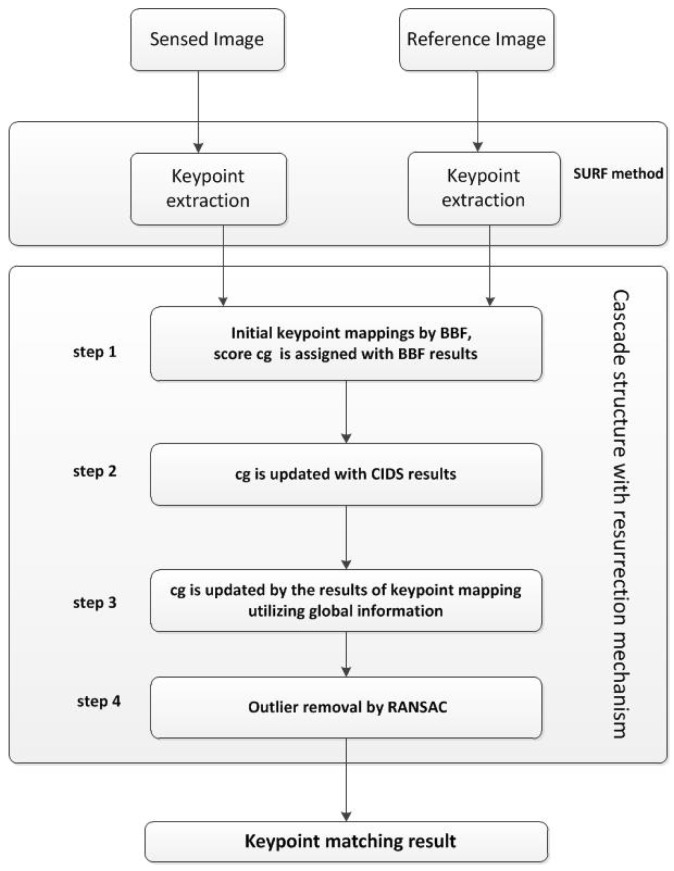
The proposed cascade structure with the resurrection mechanism.

To apply the proposed resurrection mechanism to build more accurate keypoint mappings on multispectral images, we designed a cascade structure including four steps to remove outlier keypoint mappings. Firstly, the SURF keypoints and descriptors are extracted from both the reference and the sensed image. Note, other techniques, such as SIFT, can be applied here. The keypoints and descriptors have a great impact on the final registration accuracy, but this paper is focused on the cascade structure. Then, the cascade is applied to remove incorrect mappings; its workflow is shown in Figure 2.

### 3.1. Initial Keypoint Mapping (Step 1)

This step aims to establish an initial set of keypoint mappings with BBF. For every keypoint $P_{r}^{i},i=1,\ldots ,N_{r}$, in the reference image *I_r_*(*x, y*), the keypoint $P_{j}^{i}$ in the sensed image *I_t_*(*x, y*) is defined to be the matched point of $P_{r}^{i}$ if the descriptor $f_{t}^{j}$ of $P_{t}^{j}$ has the smallest distance to the descriptor $f_{r}^{i}$ of $P_{r}^{i}$, namely,

(2) $$\left\|f_{r}^{i}-f_{t}^{j}\right\|<\left\|f_{r}^{i}-f_{t}^{k}\right\|,k\neq j$$

Let 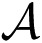 denote the set of the keypoint mappings built with Equation (2). 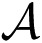 contains matches that are based on reference keypoints, *i.e.*, for every reference keypoint $P_{r}^{i}$, search the best matched test keypoint $P_{t}^{j}$ with Equation (2). Traditional BBF defines $P_{r}^{i}$ and $P_{t}^{j}$ to be matched if the following and Equation (2) hold simultaneously,

(3) $$\left\|f_{t}^{j}-f_{r}^{i}\right\|<\left\|f_{t}^{j}-f_{r}^{l}\right\|,l\neq i$$

Equations (2) and (3) say that $P_{t}^{j}$ has the smallest descriptor distance to $P_{r}^{i}$ and vice versa.

However, the matches satisfying Equations (2) and (3) may be incorrect. To understand this, suppose $f_{r}^{i}$ is the truly matched keypoint to $f_{t}^{j}$. Due to the matching ability decrease of descriptors on multispectral images, $f_{r}^{l}$ may be just marginally more close to $f_{t}^{j}$ than $f_{r}^{i}$. This discards correct matches and probably preserves incorrect matches. To cope with this issue, we wish to loosen Equation (3). For every sensed keypoint $P_{t}^{j}$, we keep *n* ≥ 1 mapping reference keypoint according to the descriptor distance. Specifically, we sort $\left\|f_{t}^{j}-f_{r}^{i}\right\|$ in an ascending order and then keep the first *n* reference keypoint $f_{r}^{i}$ as the mapping keypoint for $f_{t}^{j}$, resulting in a matching set 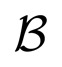. The intersection 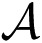 ∩ 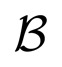 is the initial set of keypoint mappings to which Step 2 is applied. The value of *n* is typically set to three, to ensure that only the keypoint mappings meeting the BBF rule will be preserved for consideration in the following steps.

The mappings in 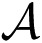 ∩ 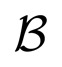 are divided into three levels. Let ${\text{P}}_{\text{r}}=\left\{P_{r}^{i}\right\}$ and ${\text{P}}_{\text{t}}=\left\{P_{t}^{j}\right\}$ denote the set of reference and test keypoints. For a mapping ${\text{P}}_{\text{r}}^{i_{0}}∼{\text{P}}_{\text{t}}^{j_{0}},P_{r}^{i_{0}}\in {\text{P}}_{\text{r}},P_{t}^{j_{0}}\in {\text{P}}_{\text{t}}$, by the rule of Step 1, $P_{t}^{j_{0}}$ is the closest keypoint for $P_{r}^{i_{0}}$, and $P_{r}^{i_{0}}$ is one of the *n* ≥ 1 closest keypoints for $P_{t}^{j_{0}}$. The score for the mapping $P_{r}^{i_{0}}∼P_{t}^{j_{0}}$ is determined as follows. If $P_{r}^{i_{0}}$ is the first closest keypoint for $P_{t}^{j_{0}}$, then *cg* is set to three; if $P_{r}^{i_{0}}$ is the second closest keypoint for $P_{t}^{j_{0}}$, *cg* is set to two; if $P_{r}^{i_{0}}$ is the third closest keypoint for $P_{t}^{j_{0}}$, *cg* is set to one.

### 3.2. Color and Intensity Distribution of Segment (Step 2)

**Figure 3:**
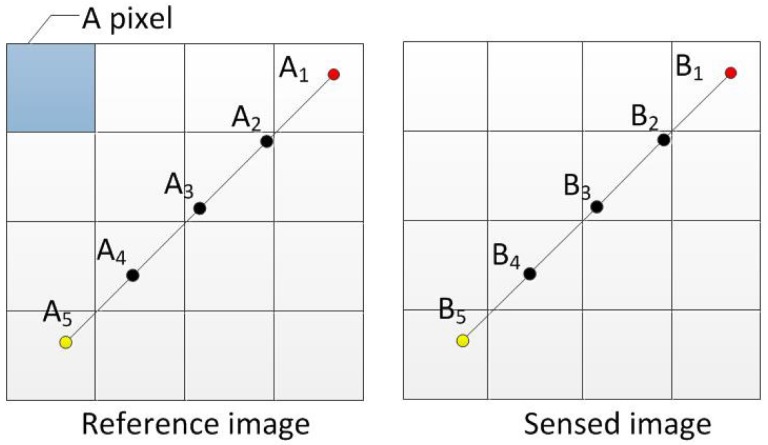
Illustration of sampling the two segments *A*_1_*A*_5_ and *B*_1_*B*_5_.

This step follows the idea of CIDS (color and intensity distribution of segment) [21]. The correlation of pixel color/gray values on two segments characterizes the quality of the two pairs of keypoint mappings *(A*_1_ ∼ *B*_1_) and *(A*_5_ ∼ *B*_5_) in Figure 3. To compute the correlation, a sampling process is conducted along the segment *A*_1_*A*_5_ and *B*_1_*B*_5_, giving two arrays of the same number of entries. The color values of the sampled points are generated with a bilinear interpolation.

Next, the two arrays of sampled points *(A*_1_*, A*_2_, *A*_3_, *A*_4_, *A*_5_) and (*B*_1_, *B*_2_, *B*_3_, *B*_4_, *B*_5_) are normalized to obtain x = {*x*_1_, *x*_2_, *x*_3_, *x*_4_, *x*_5_} and y = {*y*_1_, *y*_2_, *y*_3_, *y*_4_, *y*_5_}, such that ‖x‖ = ‖y‖ = 1.

Then, a *L*_2_-norm *d* is computed in Equation (4) to measure the similarity between the segment *A*_1_*A*_5_ and *B*_1_*B*_5_. Formally,

(4) $$d=\left\|\text{x}-\text{y}\right\|=\sqrt{\sum_{i=1}^{n}{\left(x_{i}-y_{i}\right)}^{2}}$$

where x and y represent the two normalized arrays and *n* represents the number of the sampled points. Ideally, *d* = 0 when the keypoint mappings (*A*_1_ ∼ *B*_1_) and (*A*_5_ ∼ *B*_5_) are infinitely accurate and *I_r_*(*x, y*) and *I_t_*(*x, y*) are completely identical under a certain transformation.

Since ‖x‖ = ‖y‖ = 1, by triangle inequality ‖x − y‖ ≤ ‖x‖ + ‖y‖ = 2. If *d* is greater than a predetermined threshold *t*, the two keypoint mappings will be classified as incorrect. A loose (*i.e.*, big) threshold *t* is set here, so that only the keypoint mappings between which the two segments differ significantly will be removed.

The detailed implementation applies a voting scheme as follows.

1. Choose two mappings randomly;
2. Calculate *d* with Equation (4) for the two mappings. If *d* is smaller than a preset threshold *t*, vote for both mappings, otherwise vote against them;
3. Repeat 1 and 2 until all such two keypoint mappings are considered;
4. Count the votes for every keypoint mapping *N*, and score it with:
  (5) $$cg_{t}=\{\begin{array}{ll} 3 & N\geq 0.6\cdot \left(N_{m}-1\right)\\ 2 & 0.5\cdot \left(N_{m}-1\right)\leq N<0.6\cdot \left(N_{m}-1\right)\\ 1 & 0.4\cdot \left(N_{m}-1\right)\leq N<0.5\cdot \left(N_{m}-1\right)\\ 0 & N<0.4\cdot \left(N_{m}-1\right) \end{array}$$
  where *N_m_* is the number of all keypoint mappings input to Step 2.

Utilize Equations (1) and (5); the value of *cg_c_* is updated by Equation (6),

(6) $$cg_{c}=\{\begin{array}{ll} 3 & N\geq 0.6\cdot \left(N_{m}-1\right),cg_{p}>1\\ 2 & 0.5\cdot \left(N_{m}-1\right)\leq N<0.6\cdot \left(N_{m}-1\right),cg_{p}>1\\ 2 & N\geq 0.6\cdot \left(N_{m}-1\right),cg_{p}=1\\ 1 & 0.4\cdot \left(N_{m}-1\right)\leq N<0.5\cdot \left(N_{m}-1\right),cg_{p}>1\\ 1 & 0.5\cdot \left(N_{m}-1\right)\leq N<0.6\cdot \left(N_{m}-1\right),cg_{p}=1\\ 0 & \text{others} \end{array}$$

In our experiments, *t* is set to 0.6, since the difference of x and y can be large on multispectral images. After this step, we obtain a set of keypoint mappings 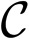.

### 3.3. Evaluate Keypoint Mappings with Global Information (Step 3)

In this step, we utilize global information to search “good” mappings in 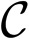. Consider three keypoint mappings in 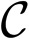, $\left(K_{t}^{i_{1}},K_{r}^{j_{1}}\right)$, $\left(K_{t}^{i_{2}},K_{r}^{j_{2}}\right)$ and $\left(K_{t}^{i_{3}},K_{r}^{j_{3}}\right)$. They determine an affine transformation *T*. If all three mappings are correct, *T* is close to the ground truth, so the similarity metric between reference *I_r_*(*x, y*) and the transformed test $I_{t}^{T}\left(x,y\right)$ is high. This work employs the number of overlapped edge pixels (NOEP) [25] as the similarity metric.

(7) $$S\left(I_{r}\left(x,y\right),I_{t}^{T}\left(x,y\right)\right)=\sum E_{r}\left(x,y\right)\cdot E_{t}^{T}\left(x,y\right)$$

where *E_r_*(*x, y*) and $E_{t}^{T}\left(x,y\right)$ are the binary edge maps of *I_r_*(*x, y*) and $I_{t}^{T}\left(x,y\right)$.

**Figure 4:**
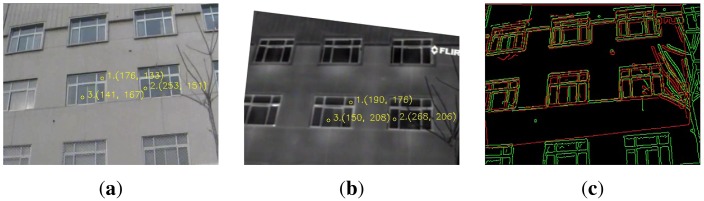
(**a**) *I_r_*(*x, y*); (**b**) , $I_{t}^{T}\left(x,y\right)$, illustrating three keypoint mappings that are to be assessed. The three mappings determine an affine transformation *T*, and then, we compute the similarity metric between *I_r_*(*x, y*) and $I_{t}^{T}\left(x,y\right)$ with Equation (7); (**c**) Overlapped edge maps, showing the overlapped edge maps where green pixels are from visible image *I_r_*(*x, y*) and red pixels are from IR image $I_{t}^{T}\left(x,y\right)$. When *T* is close to the ground truth, a majority of edge pixels is expected to be overlapped, resulting in a high similarity metric.

Figure 4 illustrates an example of utilizing the global information. Three keypoint mappings determine a transformation *T* shown in Figure 4a,b. If *T* is close to the ground truth, the similarity metric between *I_r_*(*x, y*) and $I_{t}^{T}\left(x,y\right)$ is high, *i.e.*, the number of overlapped edge pixels shown in Figure 4c is large. Consequently, the quality of three keypoint mappings can be evaluated with their determined *T* through the similarity $S\left(I_{r}\left(x,y\right),I_{t}^{T}\left(x,y\right)\right)$. Note, this step can be easily adapted to four or more keypoint mappings to account for projective or polynomial transformations. Additionally, the similarity metric in Equation (7) can be substituted for other metrics, such as mutual information (MI) [17]. In practice, Equation (7) performs as well as or slightly better than MI and requires less computational cost.

There are multiple combinations of three keypoint mappings, and a keypoint mapping appears in multiple triplets, which yield different similarity metrics. Therefore, an iterative process is utilized to find the maximum similarity metric for every keypoint mapping. The iterative process outputs the maximum similarity for a keypoint mapping over all triplets in which it appears. Formally, the maximum similarity metric by $\left(K_{t}^{i_{1}},K_{r}^{j_{1}}\right)$ is defined as:

(8) $$S_{\max}\left(K_{t}^{i_{1}},K_{r}^{j_{1}}\right)=\underset{\left(K_{t}^{i_{2}},K_{r}^{j_{2}}\right),\left(K_{t}^{i_{3}},K_{r}^{j_{3}}\right)}{\max}S\left(I_{r}\left(x,y\right),I_{t}^{T}\left(x,y\right)\right)$$

where *T* is determined by $\left(K_{t}^{i_{1}},K_{r}^{j_{1}}\right)$, $\left(K_{t}^{i_{2}},K_{r}^{j_{2}}\right)$, $\left(K_{t}^{i_{3}},K_{r}^{j_{3}}\right)$.

The iterative process outputs the maximum similarity metric for each keypoint mapping. $S_{\max}\left(K_{t}^{i_{1}},K_{r}^{j_{1}}\right)$ measures the quantity of image content that can be brought into alignment by $\left(K_{t}^{i_{1}},K_{r}^{j_{1}}\right)$. A larger *S*_max_ represents that the mapping $\left(K_{t}^{i_{1}},K_{r}^{j_{1}}\right)$ is better from the view of the entire image content. We preserve the top mappings of 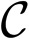 ranked according to the *S*_max_, resulting in a set of mappings 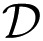 to be processed in the next step. The detailed scoring rules are shown in Equation (9), where *MAXS* is the maximum value in all *S_max_*. 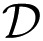 can also serve as the keypoint mapping of good quality for fast sample consensus [23].

(9) $$cg_{t}=\{\begin{array}{ll} 3 & S_{\max}\geq 0.95\cdot \text{MAXS}\\ 2 & 0.90\cdot \text{MAXS}\leq S_{\max}<0.95\cdot \text{MAXS}\\ 1 & 0.85\cdot \text{MAXS}\leq S_{\max}<0.90\cdot \text{MAXS}\\ 0 & S_{\max}<0.90\cdot <\text{MAXS} \end{array}$$

Utilize Equations (1) and (9); the value of *cg_c_* is updated by Equation (10),

(10) $$cg_{c}=\{\begin{array}{ll} 3 & S_{\max}\geq 0.95\cdot \text{MAXS},cg_{p}>1\\ 2 & 0.90\cdot \text{MAXS}\leq S_{\max}<0.95\cdot \text{MAXS},cg_{p}>1\\ 2 & S_{\max}\geq 0.95\cdot \text{MAXS},cg_{p}=1\\ 1 & 0.85\cdot \text{MAXS}\leq S_{\max}<0.90\cdot \text{MAXS},cg_{p}>1\\ 1 & 0.90\cdot \text{MAXS}\leq S_{\max}<0.95\cdot \text{MAXS},cg_{p}=1\\ 0 & \text{others} \end{array}$$

### 3.4. RANSAC (Step 4)

At the last step, we apply the RANSAC algorithm, because there are still some outlier keypoint mappings after Step 3, although most of the incorrect keypoint mappings are expected to have been removed. Random sample consensus (RANSAC) is an iterative approach to estimating the parameters of a mathematical model from a set of observed data containing outliers. RANSAC performs well in removing outliers of keypoint mappings if the correct ratio is high. However, the performance of RANSAC decreases dramatically especially when the correct ratio is low, e.g., 20% or less. Due to this, not all of the keypoint mappings built with the first three steps are used as the input of Step 4; rather, only the keypoint matches whose *cg* are two or three after Step 3 are fed into Step 4, since these keypoint matches have a greater probability of being correct.

Affine or projective transformations are utilized with RANSAC to remove outliers. When the distance of real scene content to the camera is the same, an affine transformation would be enough to account for the misalignment. When the distance varies from point to point, a projective transformation or polynomial transformation is necessitated. Polynomial transformations require at least six keypoint mappings, which significantly increases the possibility that a sample composed of six mappings contains incorrect ones. Consequently, projective transformations are utilized to address images of scene depth, and the proposed method can build correct mappings (see Figure 8 and its associated text).

Table 1 gives the experimental results. The datasets ‘Country’ to ‘Water’ are multispectral images, but the spectral distance is small (about 300 nm). On these datasets, the SIFT descriptor yields a high correct ratio of keypoint mappings, and therefore, RANSAC can further effectively remove incorrect keypoint mappings. The dataset ‘EOIR’ has more multimodality, and the correct ratio of initial keypoint mappings on it is markedly lower. Consequently, it becomes a challenging problem for RANSAC to preserve the correct mappings. See Section 4 for a detailed analysis.

## 4. Experimental Results

To investigate the performance of the proposed method, many experiments were conducted. Images include the data from the reference [26], ‘EOIR’ data and the dataset ‘VS_LWIR’from [1]. The dataset [26] includes 477 image pairs in nine categories taken with RGB and NIR (near-infrared). Dataset ‘EOIR’ includes 87 image pairs captured by ourselves, 12 Landsat image pairs from NASA, four remote sensing image pairs of the 2008 Sichuan earthquake and two image pairs from the OSUColor and Thermal Database. The 87 image pairs include indoor scenes and outdoor scenes, with one image taken with visible light and the other taken with middle-wave infrared (MWIR) light. In addition to the spectral distance, they are taken at different times, so the content of one image may be slightly different from that of the other. The 12 Landsat image pairs are downloaded from [27] with one taken with the visible band, e.g., Landsat 8 Band 3 Visible (0.53–0.59 μm), and the other taken with middle-wave light or the Thermal Infrared Sensor (TIRS), e.g., Landsat 8 Band 10 TIRS 1 (10.6–11.19 μm). The four remote sensing image pairs were taken over Wenchuan county (Sichuan Province, China) during the 2008 Sichuan earthquake. They were acquired by the Formosat-2 satellite. One image is a multispectral image (1960 × 1683) before the earthquake, and the other is a panchromatic image (1968 × 1705) after the earthquake of the same area. In order to further verify the performance of the proposed method for multispectral images taken at different times, we take two image pairs from the OSU Color and Thermal Database (Data 03, [28]). The two image pairs are captured by a thermal sensor (Raytheon PalmIR 250D, 25-mm lens) and a color sensor (Sony TRV87 Handycam). Dataset ‘VS_LWIR’ [1] contains 100 image pairs, one image taken with the visible bandwidth (0.4–0.7 μm) and the other taken with the long-wave infrared bandwidth (LWIR, 8–14 μm).

Dataset ‘EOIR’ is much more challenging than the dataset from [26], since a visible image has less common information with a middle-wave infrared image than a near-infrared image. Dataset ‘VS-LWIR’ is even more challenging due to a larger spectral difference. Note that not all visible and TIRS (MWIR) Landsat image pairs available at [27] are so hard as ‘EOIR’ to be registered. Some such image pairs are very close to single-spectrum pairs in terms of intensity/gradient pattern, although they are taken by different spectral light. This work aims to investigate the performance of different methods for which the multimodality is relatively strong, *i.e.*, the local gradient pattern does not match well, and the 12 Landsat image pairs have this property.

### 4.1. The Effectiveness of the Resurrection Mechanism

Firstly, we conduct experiments to evaluate the performance of the resurrection mechanism. The number of pending keypoint mappings and the number of resurrected mappings are counted. As above, the keypoint mappings are divided into three levels based on the calculated *cg_c_*. At the beginning (first) step, pending keypoint mappings are not available, and so, the resurrection mechanism is not used. At the end (fourth) step, it is not used either, as only the keypoint mappings with *cg* = 2 and *cg* = 3 are input, while the keypoint mappings with *cg* = 1 are completely discarded. Consequently, we consider the number of pending keypoint mappings in Steps 1 and 2 and the number of resurrected keypoint mappings in Steps 2 and 3.

**Figure 5:**
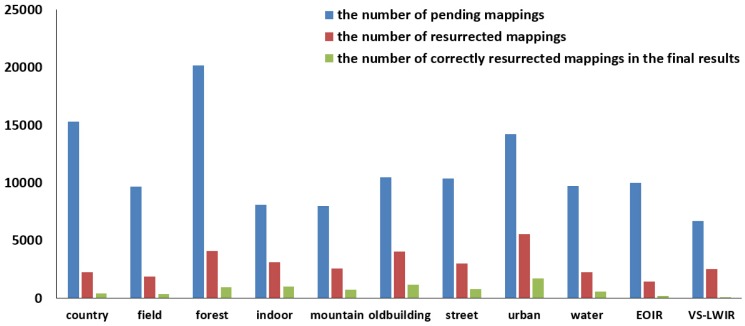
On each dataset, from left to right: the number of pending mappings, the number of resurrected mappings and the number of resurrected correct mappings.

Figure 5 shows the performance of the resurrection mechanism. From left to right are the total number of pending mappings in Steps 1 and 2 (blue bar), the total number of resurrected mappings in Steps 2 and 3 (red bar) and the number of resurrected correct mappings (green bar). From Figure 5, it can be seen that the resurrection mechanism successfully recovers some wrongly-discarded correct mappings (green bar) on all datasets. In particular, the dataset ‘EOIR’ is more challenging than other datasets, as the image pairs contain much fewer correct mappings, and hence, the number of recovered correct ones by the resurrection mechanism is much smaller, as well.

### 4.2. Visual Results

**Figure 6:**
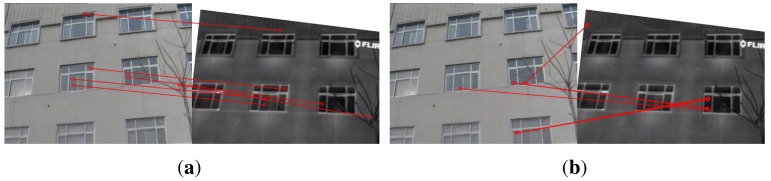
Matching result on an image pair from ‘EOIR’ taken by ourselves. The infrared image is transformed to investigate the matching performance under rotation. (**a**) The proposed method performs well under rotation; and (**b**) SIFT + RANSAC yields some incorrect matches due to the repeating windows of buildings. Such incorrect mappings are very difficult to remove, since the local gradient patterns (descriptors) of the windows lying in different position are similar to each other.

Figure 6 gives the visual result of keypoint mappings built with the proposed method and SIFT + RANSAC on an image pair from ‘EOIR’. Visually, the proposed method builds six correct keypoint matches, while SIFT + RANSAC gives two correct mappings. The incorrect mappings by SIFT + RANSAC are difficult to remove, since they are caused by the repeating structure and the distinctiveness decrease of descriptors on multispectral images.

**Figure 7:**
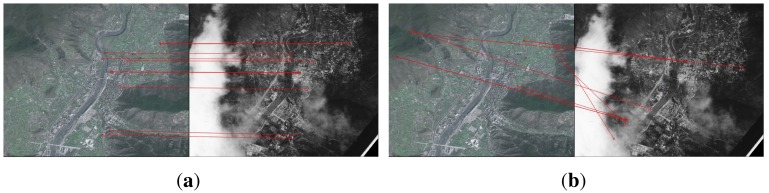
Matching result on a remote sensing image pair taken during the 2008 Sichuan earthquake (on a scale of 1:10,000) from the dataset ‘EOIR’. (**a**) The proposed method; (**b**) SIFT + RANSAC. The clouds appearing in the IR image do not generate incorrect matches for the proposed method, since they have been removed step by step in the cascade structure. SIFT + RANSAC barely generates a keypoint mapping due to the lack of texture in the cloud area (hence, fewer keypoints). The repeating structure of image content causes mismatches for SIFT + RANSAC.

Figure 7 gives another visual result of keypoint mappings on an image pair taken during the 2008 Sichuan earthquake from the dataset ‘EOIR’, with all five mappings built with SIFT + RANSAC incorrect in Figure 7b. In the proposed method, more than one mapping reference keypoint is assigned to a test keypoint in the first step, which helps preserve more keypoint mappings. Furthermore, clouds appear in the IR image, which cause occlusion and mismatches. The proposed method utilizes the descriptors, as well as complementary information, providing six correct keypoint matches in this image pair in Figure 7a.

**Figure 8:**
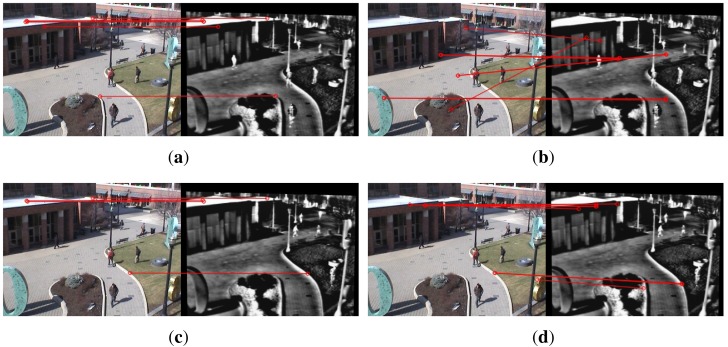
Matching results on two image pairs from the OSUColor and Thermal Database. (**a**) Proposed method; and (**c**) proposed method results; (**b**) SIFT + RANSAC; and (**d**) SIFT + RANSAC results. The top two images were taken at the same time; the bottom were taken at different times. Although this image pair is multispectral, its property in some local area is close to being single-spectrum. For example, the house ceiling is brighter than other areas on both visible and IR images, which means the similarity of the local pattern. SIFT + RANSAC contain some correct matches.

Figure 8 shows the matching results on image pairs from the OSU Color and Thermal Database. One image pair is taken at the same time, while the other is taken at different times. The proposed method established six correct mappings on Figure 8a,c, indicating that the effect of imaging time on the accuracy of keypoint mapping is small. It is interesting to see that SIFT + RANSAC performs better in Figure 8d, taken at different times than in Figure 8b. The reason may be that although the image pairs shown in Figure 8d,c are taken at different times, their scene does not change much, and they are still of the same resolution. Again, these mappings are difficult to remove, since the local gradients encoded by descriptors are very similar to each other.

The proposed method also outperforms SIFT [4] GS_SIFT [29], ISS [30], ORB[31] and FREAK [32]; however, the visual results are not listed here, and only the results of the proposed and SIFT + RANSAC are given. See the following quantitative analysis in Table 1.

### 4.3. Quantitative Results

This section presents the quantitative results. We compare the proposed method with the descriptors SIFT, GS_SIFT, ISS, ORB, FREAK and SIFT + RANSAC. The distances between keypoints are calculated for every keypoint mapping, and we count the number of keypoint mappings with distance falling in a range. Then, the histogram of the calculated distances is generated for every dataset with three bins [0, 2], [2, 5] and > 5. Table 1 gives the number histograms for which each bin counts the number of keypoint mappings with the distance falling in the bin. For example, on the dataset ‘Country’, 898 keypoint mappings built with the proposed method have a distance less than two; 99 keypoint mappings have a distance lying in [2, 5]; and nine keypoint mappings have a distance greater than five. On ‘Country’, SIFT + RANSAC gives 847 mappings of a distance less than two, 182 mappings of a distance less than 0.15 and 195 mappings of a distance larger than 0.16.

ISS is not only an approach to extracting descriptors, but one to building keypoint mappings by finding the rough orientation difference between two images. SIFT, GS_SIFT, ORB and FREAK are mapped with BBF in our experiment. These keypoints and descriptors are detected with the source code in OpenCV, and the mapping keypoints are implemented with OpenCV. For all of these methods, OpenCV basically follows the parameter setups of the original papers.

The total number of keypoint mappings built by different methods varies from one to another, so we assess the performance in terms of the percent of correct and incorrect mappings. Table 1 also gives the percent histogram of the distance between mapped keypoints. For example, on dataset ‘Country’, the proposed method has 89% (0.89) of keypoint mappings with a distance falling in [0, 2] and 10% (0.10) falling in [2, 5], while SIFT + RANSAC has 69% (0.69) in [0, 2] and 15% (0.15) in [2, 5] and ISS has 19% and 5% falling in [0, 2] and [2, 5]. The datasets ‘Country’ to ‘Water’ include multispectral image pairs, but the spectral distance is small (300 nm–400 nm); therefore, their characteristics are very close to monomodal image pairs. SIFT or SURF can build keypoint mappings of a sufficiently high correct ratio, and accordingly, SIFT + RANSAC performs relatively well. On datasets ‘Country’, ‘Field’, ‘Forest’, ‘Indoor’, ‘Mountain’, ‘Oldbuilding’, ‘Street’, ‘Urban’ and ‘Water’, SIFT provides correct ratios of 50% (0.50), 68% (0.68), 75% (0.75), 75% (0.75), 67% (0.67), 86% (0.86), 72% (0.72), 96% (0.96) and 71% (0.71).

Dataset ‘EOIR’ includes image pairs taken by visible light and middle/long-wave infrared light and is much more challenging, since the spectral distance is larger. On this dataset, the presented method has 30% (0.30) and 53% (0.53) keypoint mappings with a distance falling in [0, 2] and [0, 5], while SIFT + RANSAC has 9% (0.09) and 16% (0.16) in [0,2] and [0, 5], SIFT has 10% (0.10) and 13% (0.13), GS_SIFT has 9% (0.09) and 12% (0.12), ISS has 7% (0.07) and 11% (0.11), ORB has 6% (0.06) and 8% (0.02) and FREAK has 3% (0.03) and 6% (0.06). Dataset ‘VS-LWIR’ is even more challenging than ‘EOIR’, as it can be seen that the correct rates for all methods are lower than on ‘EOIR’. For example, the presented method has 15% (0.15) and 48% (0.48) on ‘VS-LWIR’, and SIFT + RANSAC has only 2% (0.02) and 6% (0.06).

RANSAC does not improve much over the original SIFT, since the keypoint mappings built with the original SIFT contain too many incorrect ones, which makes RANSAC fail to identify correct mappings. Compared with other methods, the presented method increases the correct ratio. Since the dataset is relatively large, this result shows that the proposed method is more suitable for multispectral images than other methods.

## 5. Conclusions

With the insight of the boosting technique, we proposed a cascade structure of four steps with a resurrection mechanism for registering multispectral images. The cascade structure classifies keypoint mappings at a step to be correct, incorrect and pending. Unlike the traditional approaches that typically determine keypoint mappings to be correct or not with a one-pass process, the “pending” keypoint mappings will be determined by the rules at subsequent steps. This effectively overcomes the drawback of applying a single algorithm for registering images. Furthermore, the resurrection mechanism gets back some of the correct mappings that were misclassified at previous steps. The experimental results demonstrate that the cascade structure can provide more robust registration than the state-of-the-art.

Several directions are possible in the future. Improving the keypoint repeatability and the repeatability and distinctiveness of descriptors are the building blocks for keypoint mappings on multispectral images. At each step, different approaches can be applied to investigate the performance of the cascade, and the number of steps can be adjusted for applications. Last, but not least, other image information, such as CIDS, can be adapted to multispectral images to supplement the matching ability of the descriptors.

## Figures and Tables

**Table 1:** The error distribution histogram.

		**Country**			**Field**			**Forest**			**Indoor**			**Mountain**			**Oldbuilding**			**Street**			**Urban**			**Water**			**EOIR**			**VS-LWIR**	
ERROR	[0, 2]	[2, 5]	> 5	[0, 2]	[2, 5]	> 5	[0, 2]	[2, 5]	> 5	[0, 2]	[2, 5]	> 5	[0, 2]	[2, 5]	> 5	[0, 2]	[2, 5]	> 5	[0, 2]	[2, 5]	> 5	[0, 2]	[2, 5]	> 5	[0, 2]	[2, 5]	> 5	[0, 2]	[2, 5]	> 5	[0, 2]	[2, 5]	> 5
CASCADE	898	99	9	2540	145	51	4749	18	0	5641	96	2	5641	96	2	6987	75	0	3502	124	0	12,717	19	0	1777	84	17	162	130	257	78	117	320
	0.89	0.10	0.01	0.93	0.05	0.02	0.99	0.01	0.00	0.98	0.02	0.00	0.98	0.02	0.00	0.99	0.01	0.00	0.97	0.03	0.00	0.998	0.002	0.00	0.95	0.04	0.01	0.30	0.23	0.47	0.15	0.23	0.62
SIFT + RANSAC	847	182	195	2431	274	164	3635	561	31	5470	614	29	6474	600	106	8004	590	3	4055	527	16	14,412	825	1	1971	230	170	75	54	700	15	29	744
	0.69	0.15	0.16	0.85	0.10	0.05	0.86	0.13	0.08	0.89	0.10	0.01	0.90	0.08	0.02	0.93	0.07	0.00	0.88	0.11	0.01	0.95	0.05	0.00	0.83	0.10	0.07	0.09	0.07	0.84	0.02	0.04	0.94
SIFT	379	87	285	691	106	226	1244	276	140	464	23	128	742	258	110	696	81	28	356	94	43	735	8	23	425	44	129	22	7	202	2	11	3948
	0.50	0.12	0.38	0.68	0.10	0.22	0.75	0.17	0.08	0.75	0.04	0.21	0.67	0.23	0.10	0.86	0.10	0.03	0.72	0.19	0.09	0.96	0.01	0.03	0.71	0.07	0.22	0.10	0.03	0.87	0.00	0.01	0.99
GS_SIFT	381	88	290	694	121	227	1254	299	144	466	24	127	753	258	111	700	81	32	362	99	43	746	8	23	431	46	132	22	7	203	2	10	4939
	0.50	0.12	0.38	0.67	0.12	0.22	0.74	0.18	0.08	0.76	0.04	0.21	0.67	0.23	0.10	0.86	0.10	0.04	0.72	0.20	0.09	0.96	0.01	0.03	0.71	0.08	0.22	0.09	0.03	0.88	0.00	0.01	0.99
ISS	213	54	884	246	73	794	639	167	1000	299	26	178	179	98	200	281	32	58	179	54	300	389	14	74	263	35	542	25	14	313	2	16	4578
	0.19	0.05	0.77	0.22	0.07	0.71	0.35	0.09	0.55	0.59	0.05	0.35	0.38	0.21	0.42	0.76	0.09	0.16	0.34	0.10	0.56	0.82	0.03	0.16	0.31	0.04	0.65	0.07	0.04	0.89	0.00	0.01	0.99
ORB	195	95	6860	295	168	2919	481	165	869	399	57	634	269	186	379	304	103	58	209	144	790	366	17	18	300	122	2501	328	87	4765	2	14	10,249
	0.03	0.01	0.96	0.09	0.05	0.86	0.32	0.11	0.57	0.37	0.05	0.58	0.32	0.22	0.45	0.65	0.22	0.12	0.18	0.13	0.69	0.91	0.04	0.04	0.10	0.04	0.86	0.06	0.02	0.92	0.00	0.00	1.00
FREAK	12	10	9567	11	9	3982	84	34	14,647	69	34	5490	97	13	5798	43	24	6864	37	6	6295	111	34	9779	32	20	6932	11	11	371	1	3	575
	0.00	0.00	1.00	0.00	0.00	1.00	0.01	0.00	0.99	0.01	0.01	0.98	0.02	0.00	0.98	0.01	0.00	0.99	0.01	0.00	0.99	0.01	0.00	0.99	0.00	0.00	0.99	0.03	0.03	0.94	0.00	0.01	0.99
